# Case of Excision of the Whole of the Genital Organs

**Published:** 1847-09

**Authors:** E. W. H. Beck

**Affiliations:** Asst. Surg. U. S. A.


*Gase of Excision of the whole of the Genital Organs.*—By E. W. H. Beck, Asst. Surg. U. S. A. (Communicated in a letter to the Editor, dated, U. S. Hospital, *Matamoras, Mexico*, May 8, 1847.)—Sir.—Permit me to report to you a case that lately came under my care, which, from its oddity and interest, is certainly entitled to a page in your excellent journal. J. B., ætat. 31, stout build, bilious temperament, had been a member of the army, but now in the position of bar-beeper in a grocery; on the evening of March 9th, during a fit of delirium tremens, and unmanageable behaviour, was confined in the guard-house. A few minutes after his confinement he borrowed of a fellow-prisoner a short, thick, one-bladed, pocket knife, with which he completely excised the whole of the genital apparatus, close to the body. Flinging them violently into one corner of the room, he very heroically remarked—"Any d—d fool can cut his throat, but it takes a soldier to cut his privates off." This was at seven and a half o'clock. His companion gave the alarm, and the surgeon of the Mississippi regiment, happening to be in the same building, got to him about ten minutes after the accident. Every effort was made to secure the spermatic arteries, but their immediate retraction was so great that he failed in getting them. I was sent for in consultation, but being absent from my room at the time, the courier returned to the doctor stating the fact. The man was bleeding to death, and, in the desperation of the moment, he determined to apply the actual cautery to the bleeding surface.

At eight o'clock, a few minutes after its application, I saw him, and as the bleeding had almost entirely ceased, a large bunch of rags was applied as a compress, and secured by the appropriate bandages; his hips a little elevated; cold applications to the abdomen, and perfect rest and quiet for three hours; at which time he was removed to the General Hospital, and placed in the surgical ward under my care. The amount of blood lost was estimated by all present at near or quite one gallon. One fact worthy of notice here is, that eight or ten minutes after the bleeding commenced, complete consciousness was restored, nor did he exhibit a symptom of *delirium tremens*, afterwards. On my visit next morning, he lamented his condition as a sensible man, asked my opinion of his danger; complained of no pain ; skin cool ; pulse slightly jerking, and tremulous ; 88 in frequency. I ordered him barley water, and, fearful of hemorrhage, did not disturb the bandages until a disposition to pass water, which was early the following morning. To remove the dressing the more easily, a soft poultice was applied ; and after some difficulty in finding the urethra and passing the catheter, evacuated the bladder, with but a little oozing of blood from the surface and one minute artery, which I secured by torsion. Ever after this, his water passed without artificial assistance ; his pulse became equal and soft after a gentle aperient, and absolute diet. Dry dressings—until a yellowish sloughy secretion was coming off, when I washed with a solution of chlorinated soda, and applied simple cerate. The too luxuriant granulations, which soon arose, were suppressed with caustic, and the whole had kindly cicatrized in five weeks from the occurrence of the accident.

The last few days of the healing process, a large silver catheter, and afterwards, an oiled tent, was retained in the urethra, to prevent any contraction, to which there was a great tendency, and around which the orifice closed with a firm callous edge. The superior surface, or that above the urethra, presents a flat, or rather concave appearance, the posterior slightly elevated or ridge-like.—*Ibid.*

